# Disruption of Long-Term Depression Potentiates Latent Inhibition: Key Role for Central Nucleus of the Amygdala

**DOI:** 10.1093/ijnp/pyab011

**Published:** 2021-03-08

**Authors:** Donovan M Ashby, Carine Dias, Lily R Aleksandrova, Christopher C Lapish, Yu Tian Wang, Anthony G Phillips

**Affiliations:** 1 Hotchkiss Brain Institute, University of Calgary, Calgary, AB, Canada; 2 Department of Psychiatry, University of British Columbia, Vancouver, BC, Canada; 3 Department of Psychiatry, Djavad Mowafaghian Centre for Brain Health, University of British Columbia, Vancouver, BC, Canada; 4 Department of Psychology, Indiana University - Purdue University Indianapolis, Indianapolis, IN, United States; 5 Department of Medicine, Djavad Mowafaghian Centre for Brain Health, University of British Columbia, Vancouver, BC, Canada

**Keywords:** Synaptic plasticity, cognitive flexibility, long-term depression (LTD), central amygdala (CeA), latent inhibition (LI)

## Abstract

**Background:**

Latent inhibition (LI) reflects an adaptive form of learning impaired in certain forms of mental illness. Glutamate receptor activity is linked to LI, but the potential role of synaptic plasticity remains unspecified.

**Methods:**

Accordingly, the present study examined the possible role of long-term depression (LTD) in LI induced by prior exposure of rats to an auditory stimulus used subsequently as a conditional stimulus to signal a pending footshock. We employed 2 mechanistically distinct LTD inhibitors, the Tat-GluA2_3Y_ peptide that blocks endocytosis of the GluA2-containing glutamate α-amino-3-hydroxy-5-methyl-4-isoxazolepropionic acid receptor, or the selective glutamate n-methyl-d-aspartate receptor 2B antagonist, Ro25-6981, administered prior to the acquisition of 2-way conditioned avoidance with or without tone pre-exposure.

**Results:**

Systemic LTD blockade with the Tat-GluA2_3Y_ peptide strengthened the LI effect by further impairing acquisition of conditioned avoidance in conditional stimulus-preexposed rats compared with normal conditioning in non-preexposed controls. Systemic Ro25-6981 had no significant effects. Brain region–specific microinjections of the Tat-GluA2_3Y_ peptide into the nucleus accumbens, medial prefrontal cortex, or central or basolateral amygdala demonstrated that disruption of glutamate α-amino-3-hydroxy-5-methyl-4-isoxazolepropionic acid receptor endocytosis in the central amygdala also potentiated the LI effect.

**Conclusions:**

These data revealed a previously unknown role for central amygdala LTD in LI as a key mediator of cognitive flexibility required to respond to previously irrelevant stimuli that acquire significance through reinforcement. The findings may have relevance both for our mechanistic understanding of LI and its alteration in disease states such as schizophrenia, while further elucidating the role of LTD in learning and memory.

**Significance Statement**

Latent inhibition (LI) is a learning phenomenon whereby prior repeated exposure to inconsequential stimuli disrupts subsequent conditioning of such stimuli with reinforcement. Glutamate receptor activity is linked to LI, but the potential role of synaptic plasticity remains unspecified. Systemic disruption of long-term depression (LTD) using an interference peptide to inhibit AMPA receptor endocytosis strengthened the LI effect by further impairing avoidance learning in rats pre-exposed to the tone stimulus compared with the non-preexposed condition. Targeted peptide microinjections to disrupt LTD, specifically in the central amygdala, replicated this potentiated LI effect. These findings are relevant to understanding the neural circuits underlying LI as well as the role of synaptic LTD in learning and memory. Given the prominence of aberrant LI in schizophrenia, these data may also have clinical relevance.

## Introduction

The flexible and efficient allocation of cognitive resources to guide behavior in a changing environment is important for survival. Latent inhibition (LI) is a learning phenomenon where repeated exposure to an inconsequential stimulus impairs the subsequent conditioning of this stimulus with reinforcement (Lubow, 1973). While learning to ignore irrelevant stimuli is considered an adaptive mechanism, pathological alteration of LI is associated with cognitive dysfunction in schizophrenia (Lubow and Weiner, 2010a).

The acquisition-failure theory of LI attributes the retarded conditioning to the pre-exposed stimulus to decreased associability (Lubow et al., 1976) or its salience (Pearce and Hall, 1980). Alternatively, the competition theory emphasizes opposing processes between 2 conflicting associations, specifically conditional stimulus (CS)-no event acquired in pre-exposure and CS-US acquired subsequently during conditioning, which compete for behavioral expression/retrieval (Weiner, 1990). Reconciliation of these 2 approaches postulates that CS pre-exposure attenuates subsequent acquisition of the CS–event association, which in turn competes with the original CS–no event association (Lubow and Weiner, 2010b). Behavioral, physiological, and pharmacological manipulations can promote or impair switching to respond according to the stimulus reinforcement association and thereby weaken or strengthen the expression of LI (Weiner and Feldon, 1997; Weiner, 2003). Many of these accounts implicate changes in synaptic plasticity in different learning processes during LI, but there are no direct tests of this hypothesis.

Descriptions of the neural underpinnings of LI traditionally focus on the nucleus accumbens (NAc) and the action of dopamine therein (Weiner and Arad, 2009). The switching model of LI (Weiner and Feldon, 1997), which implicates NAc circuits in resolving conflicts arising from 2 competing associations (CS-event/no event), is supported by an extensive literature on both the modulatory effects of dopaminergic drugs (Weiner et al., 1996; Schiller et al., 2006) and NAc lesions on aspects of LI (Weiner et al., 1996, 1999; Jongen-Rêlo et al., 2002; Pothuizen et al., 2005; Floresco, 2015). These studies show that lesions of the NAc shell can increase, whereas NAc core lesions can decrease, flexibility in LI (Weiner et al., 1996; Jongen-Rêlo et al., 2002; Gal et al., 2005). In addition, the dopamine releaser amphetamine, which can produce/exacerbate psychotic-like symptoms in both animals and humans, disrupts LI at the conditioning stage, while typical and atypical antipsychotic drugs, scopolamine, and glutamate n-methyl-d-aspartate receptors (NMDAR) antagonists (phencyclidine, ketamine, and MK-801) produce persistent LI also via effects at conditioning (Gaisler-Salomon and Weiner, 2003; Weiner, 2003; Gaisler-Salomon et al., 2008).

The use of specific inhibitors of synaptic plasticity provides an effective strategy for delineating detailed synaptic mechanisms underlying different learning paradigms (Citri and Malenka, 2008; Howland and Wang, 2008; Collingridge et al., 2010; Takeuchi et al., 2014). Long-term depression (LTD) is a form of synaptic plasticity involving activity-dependent weakening of excitatory neurotransmission at glutamate synapses. LTD is implicated in mediating cognitive and behavioral flexibility in tasks that involve a change in reinforcement contingencies, whereby previously acquired contingencies disrupt acquisition of behavioral responding guided by the current contingencies. Our group and others have shown that blocking LTD prior to conditioning impairs fear extinction (Dalton et al., 2008, 2012), spatial reversal learning (Kim et al., 2011; Dong et al., 2013), and natural forgetting (Hardt et al., 2014; Migues et al., 2016).

Based on these previous findings, we reasoned that LTD may participate in the changing reinforcement contingencies in stimulus pre-exposed animals. In accordance with the competition theory (Weiner, 1990; Weiner and Feldon, 1997), we hypothesized that disruption of LTD prior to conditioning in an LI paradigm would have a selective detrimental effect on avoidance learning in rats pre-exposed to the CS by interfering with the cognitive flexibility required to overwrite a stimulus-no response association while having no effect in the non-preexposed (NPE) group where no prior association had been acquired. In the present study, LTD was blocked by either disrupting clathrin-dependent endocytosis of GluA2-containing α-amino-3-hydroxy-5-methyl-4-isoxazolepropionic acid receptors (AMPARs), the critical final step in LTD expression, using the Tat-GluA2_3Y_ peptide (Ahmadian et al., 2004; Brebner et al., 2005; Dalton et al., 2008, 2012), or by inhibiting GluN2B subunit-containing NMDARs implicated in the initiation of LTD, using the selective antagonist Ro25-6981 (Liu et al., 2004). Initially, both LTD manipulations were administered systemically to examine the potential role of LTD in modulating LI of acquisition of 2-way active avoidance of a footshock predicted by an auditory tone stimulus. To further identify key brain regions where inhibition of AMPAR endocytosis may mediate effects of LI, the Tat-GluA2_3Y_ peptide was microinjected into the NAc and medial prefrontal cortex (mPFC), a key afferent projection to the NAc (George et al., 2010; Lingawi et al., 2016). Given our use of an avoidance paradigm to assess LI, we also included the central nucleus of the amygdala (CeA) due to its roles in active defensive responses (Tillman et al., 2018) and the control of dopaminergic activity during appetitive and aversive learning (Ahn and Phillips, 2002; Steinberg et al., 2020). A less well-known function of the CeA is in allocating attention resources to cues when their predictive value changes (Holland and Gallagher; 1993a, 2006; Lee et al., 2006). Extending this capacity to LI suggests the CeA may play a similar role when familiar cues with minimal predictive value suddenly gain significance through reinforcement.

## Methods and Materials

### Subjects

Male Sprague-Dawley rats (Charles River, Montreal, Canada) weighing 200-220 g on arrival were pair-housed in a colony room (temperature: 21°C ± 1°C) under a reverse light cycle (light off 7:00 am–7:00 pm). Food and water were available ad libitum. After the i.v. surgery, the animals were isolated for 3 days to ensure that the surgical wound on their back was sufficiently healed before being housed again with their cage mate. All experiments followed the principles of laboratory animal care and were conducted in accordance with the standards of the Canadian Council on Animal Care and the Guidelines for the Care and Use of Mammals in Neuroscience and Behavioral Research (National Research Council 2003). All the experiments were approved by the Committee on Animal Care, University of British Columbia.

### Surgery

#### Implantation of i.v. catheter

Rats were anaesthetized with isoflurane (oxygen flow rate: 2 L/min; isoflurane: induction: 4%; maintenance: 1.5–2.5%; Baxter Corporation, Canada) and an indwelling silastic catheter (Dow Corning Corporation, US) was implanted with the proximal end inserted into the right jugular vein. The distal end was connected to a plastic screw-on connector (Plastics-One Inc., Roanoke, VA, USA) mounted on a square mesh (Plastics-One Inc., US) and secured by dental cement. The catheter was located s.c. and the plastic connector exited through an incision between the scapulae. The i.v. catheter was flushed daily with a heparinized (30 IU/mL; LEO Pharma Inc., Canada) ampicillin solution (0.2 mL of 50 mg/mL; Novopharm, Canada) to prevent infection and keep the catheter patent.

#### Intracranial surgery

Rats were anaesthetized with isoflurane (oxygen flow rate: 2 L/min; isoflurane: induction: 4%; maintenance: 1.5–2.5%) and mounted in a Kopf stereotaxic frame with the incisor bar set at 3.3 mm below the interaural line. Bilateral guide cannulas (23-gauge, Small Parts Inc. Miami Lake) were implanted, aimed 1.5 mm above the NAc (anteroposterior [AP]: +1.65, mediolateral [ML]: ±1.2, dorsoventral [DV] −5.5), 1.5 mm above the central amygdala (AP: −1.9; ML: ±4, DV: −6.7), 1.5 mm above the basolateral amygdala (AP: −2.1, ML: ±4.8, DV: −6.8), or 0.5 mm above the mPFC (AP: +2.1, ML: ±0.8, DV: −2.2). AP, ML, and DV coordinates were calculated relative to Bregma (Paxinos and Watson, 2013). Injection needles extended 1.5 mm beyond the guide cannula tip for NAc, CeA, and basolateral amygdala (BLA) experiments and 0.5 mm beyond the guide cannula tip for mPFC experiments. The cannulas were anchored to the skull with dental acrylic cement (Simplex; Kent Dental Supplies, Gillingham, Kent, UK) and 4 surgical screws. Stainless-steel obturators (30-gauge, Small Parts Inc.) were inserted in the cannula to prevent occlusion.

### LI Protocol

Each avoidance apparatus (41.8 × 25.5 × 16.2 cm; Med Associates, St. Albans, VT) is comprised of 2 compartments of equal dimension separated by a guillotine door. Each compartment was equipped with 4 photocells, a wall-mounted cue light, and a white noise generator (approximately 60 dB) on each rear end. The LI protocol consisted of 2 different phases. On day 1 and 2, there were 2 groups of animals pre-exposed to the test apparatus along with either: (a) the presentation of fifty 10-second conditioned stimuli (CS: white noise presented at pseudo-random intervals (range 10–120 seconds, mean: 60 seconds) (pre-exposed rats, PE), or (b) the absence of auditory stimuli (NPE). Each session lasted 60 minutes (15 minutes habituation + 45 minutes session). On day 3, 2-way avoidance training began with a 15-minute habituation period followed by 100 CS-unconditioned stimuli footshock pairing trials presented at pseudo-random intervals. The auditory CS (10 seconds) was identical to the tone presented previously to the PE group. A footshock (2 seconds, 0.75 mA) was delivered at the end of the CS. An avoidance response was defined by movement into the opposite compartment prior to CS termination. Escape responses were defined as entry into the opposite compartment during the 2-second footshock. Movement after footshock termination constituted a response failure. The main dependent variable was the number of avoidance responses recorded during the 100-trial session.

### Drugs

The selective GluN2B subunit antagonist Ro25-6981 (Sigma- Aldrich, Oakville, Canada) was dissolved in the vehicle (2% DMSO and 0.09% isotonic saline). The Tat-GluA2_3Y_ peptide was constituted of 9 amino acids (YKEGYNVYG) (Ahmadian et al., 2004) and was attached to a HIV-1-derived Tat peptide sequence (YGRKKRRQRRR) to cross the blood brain barrier and permeate cells (Schwarze et al., 1999). The scrambled peptide Tat-GluA2_Sc_ was comprised of a scrambled sequence of the same 9 amino acids (VYKYGGYNE). Both Tat-GluA2_3Y_ and Tat-GluA2_Sc_ were synthesized in the Wang laboratory. These peptides were diluted in 0.9% sterile saline for the i.v. administration and in phosphate buffer saline (0.05 M) for the intracerebral administration.

### Drug Treatment

All drug treatments were administered exclusively on day 3 prior to the initiation of 2-way avoidance training. Control assessment of LI (NPE: n = 6; PE: n = 7) involved i.v. administration of saline 45 minutes prior the onset of avoidance training. A similar protocol was used for AMPAR endocytosis inhibitor experiments, with Tat-GluA2_3Y_ (2.25 nmol/g; NPE: n = 7, PE: n = 7) or the control peptide, Tat-GluA2_Sc_ (2.25 nmol/g; NPE: n = 12, PE: n = 13) administered i.v. 45 minutes prior to starting the training session. GluN2B antagonist experiments involved i.p. injection of 6 mg/kg of Ro25-6981 (NPE: n = 6, PE: n = 12) or vehicle (NPE: n = 8, PE: n = 7) 15 minutes before placement into the apparatus. During intracerebral microinjection experiments, injection needles (33-gauge, Small Parts Inc.) were inserted into guide cannulae. Tat-GluA2_3Y_ or Tat-GluA2_Sc_ (22.5 pmol/0.5 µL) was administered at a rate of 0.5 µL/min. At the end of the injection, the needles were left in place for 2 minutes to ensure diffusion and were then replaced by obturators. Animals received bilateral injections of 0.5 µL/side into the NAc (Tat-GluA2_3Y_ NPE: n = 7, PE: n = 7; Tat-GluA2_Sc_ NPE: n = 6, PE: n = 8), 0.75 µL/side into the mPFC (Tat-GluA2_3Y_ NPE: n = 5, PE: n = 7; Tat-GluA2_Sc_ NPE: n = 5, PE: n = 6), and 0.5 µL/side into the CeA (Tat-GluA2_3Y_ NPE: n = 6, PE: n = 8; Tat-GluA2_Sc_ NPE: n = 7, PE: n = 7). To exclude possible effects of intra-CeA Tat-GluA2_3Y_ spreading into the BLA, 2 additional groups (NPE: n = 8, PE: n = 9) were tested on acquisition of 2-way avoidance following bilateral injections of Tat-GluA2_3Y_ (0.5 µL/side) into the BLA and compared with Tat-GluA2_3Y_ CeA treatment groups.

### Histology

After completion of each experiment, animals were deeply anaesthetized with isoflurane and brains were removed and stored in 20% w/v sucrose and 4% v/v paraformaldehyde solution for at least 48 hours. Coronal sections (30 µm) were stained with Cresyl violet (Fisher Scientific, Ottawa, Canada) and examined for injection site location (supplementary Figure 1).

### Statistical Analysis

The avoidance responses were analyzed using a repeated-measures mixed general linear model (GLM) using SPSS (IBM), with 10-trial bins as the within-subject variable and exposure and drug as between-subject fixed effects. Acquisition of 2-way avoidance was indicated by a main effect of trial bins, whereas LI was indicated by a main effect of exposure. To assess the impact of drug interventions (Ro25-6981 vs vehicle, Tat-GluA2_3Y_ vs Tat-GluA2_Sc_) on LI, a significant drug by exposure interaction was followed by simple main effects comparisons (SME) between drug treatment groups in PE and NPE groups, respectively, with Bonferroni correction for multiple comparisons. Escape responses were analyzed in experiments with a significant group effect on avoidance to confirm that these effects were not explainable by a nonspecific effect on locomotor activity. Differences were considered significant when *P < *.05.

## Results

**Figure 1.**
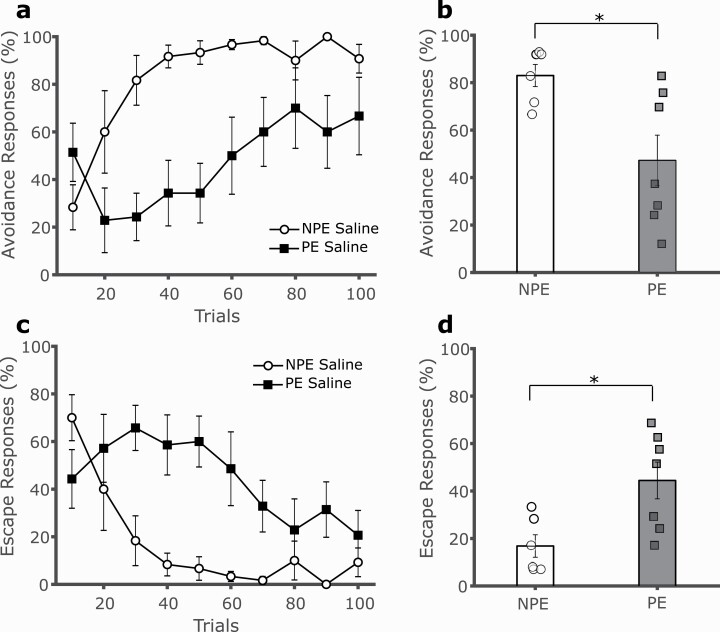
Latent inhibition (LI) of 2-way avoidance. (A) Strong LI was observed in our paradigm, as pre-exposure (PE, filled squares) to the CS significantly impaired acquisition of avoidance responding relative to non-preexposed (NPE, empty circles) rats, as observed in 10 trial bins. Successful avoidance responses involved shuttling to the opposite side of the avoidance chamber during the 10-second tone presentation, which predicted a 2-second footshock. (B) Overall avoidance percentage across 100 trials was significantly lower in PE vs NPE rats, reflecting LI. (C) Escape responses declined progressively over trials, with fewer escape responses in NPE vs PE rats. (D) Overall escape responses percentage across 100 trials was significantly lower in PE vs NPE rats. *P < .05, error bars represent SEM.

As expected, there was a significant difference in 2-way avoidance acquisition between vehicle-treated PE and NPE rats, reflecting LI. Pre-PE to the CS slowed learning of avoidance responding to subsequent CS-US pairing compared with the NPE condition (Figure 1A, B; 2-way mixed GLM, main effect of trials F(9,99) = 6.345, *P* < .001; main effect of exposure F(1,11) = 8.252, *P* = .015; trials by exposure interaction F(9,99) = 3.868, *P* < .001). Escape responses declined progressively over trials, with fewer escape responses in NPE vs PE rats (Figure 1C, D; main effect of trials F(9,99) = 5.739, *P* < .001; main effect of exposure F(1,11) = 8.408, *P* = .014; trials by exposure interaction F(9,99) = 3.752, *P* < .001).

**Figure 2.**
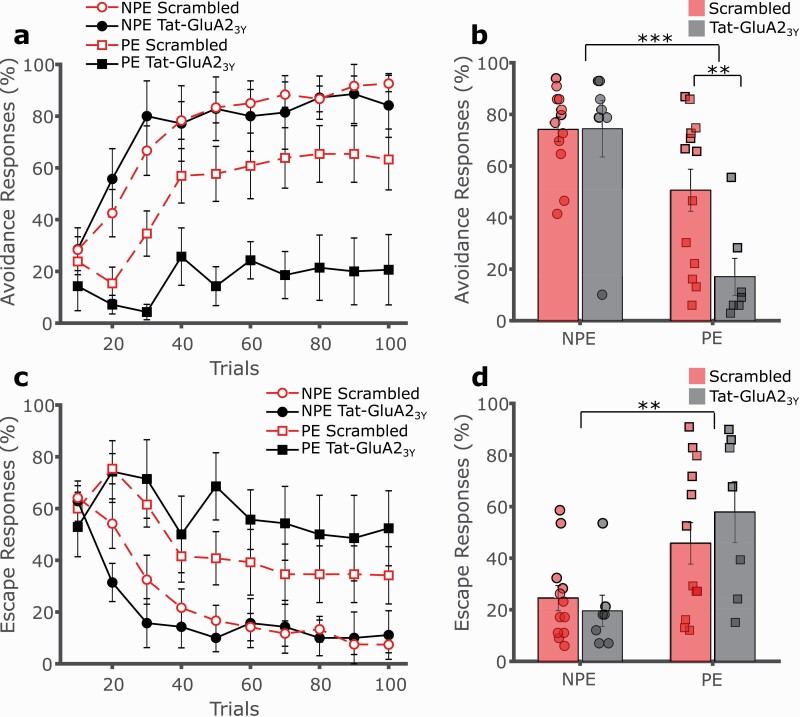
Systemic Tat-GluA23Y potentiates latent inhibition (LI) of 2-way avoidance. (A) The AMPA receptor endocytosis inhibitor Tat-GluA23Y (2.25 nmol/g, i.v., black, solid lines) was administered systemically to rats prior to conditioning. Rats pre-exposed (PE, squares) to the CS avoided significantly less than non-preexposed (NPE, circles) controls. Tat-GluA23Y markedly strengthened the LI effect by further impairing avoidance responding in PE rats (squares) relative to rats administered a scrambled control peptide (red, dashed lines), while having no effect in the NPE group (circles). (B) Overall avoidance percentage indicated a significant reduction of avoidances in Tat-GluA23Y-treated PE rats compared with the NPE group and the corresponding scrambled control, indicating a potentiation of LI. (C) Escape responses progressively declined over trials, with fewer escapes in NPE vs PE rats, although this was not observed in the PE GluA23Y group. No effect of GluA23Y treatment or interaction was observed. (D) Overall escape responses percentage was significantly lower in PE vs NPE rats, and no effect of GluA23Y treatment or interaction was observed. **P < .01, ***P < .001, error bars represent SEM.

Systemic administration of the AMPAR endocytosis interference peptide Tat-GluA2_3Y_ prior to conditioning further significantly reduced avoidance learning in PE rats relative to PE rats treated with a scrambled control peptide. Importantly, Tat-GluA2_3Y_ treatment had no measurable effect on performance in NPE rats, resulting in a much stronger LI effect (i.e., larger difference between PE and NPE groups) in the Tat-GluA2_3Y_ peptide condition (Figure 2A, B; 3-way mixed GLM, main effect of trials F(9,315) = 24.617, *P* < .001; main effect of exposure F(1,35) = 25.059, *P* < .001; main effect of drug F(1,35) = 4.256, *P* = .047; exposure by drug interaction F(1,35) = 4.365, *P* = .044; follow-up SME of drug in NPE F(1,33) = 0.001, *P* = .985; follow-up SME of drug in PE F(1,35) = 8.746, *P* = .006). Escape responses progressively declined over trials (Figure 2C, D; F(9,315) = 16.802, *P* < .001) with fewer escapes in NPE vs PE rats (F(1,35) = 13.580, *P* = .001), although this pattern was not seen in Tat-GluA2_3Y_-treated PE rats. No significant effect of Tat-GluA2_3Y_ treatment or interaction was observed (main effect of drug F(1,35) = 0.208, *P* = .651; exposure by drug interaction F(1,35) = 1.098, *P* = .302).

**Figure 3.**
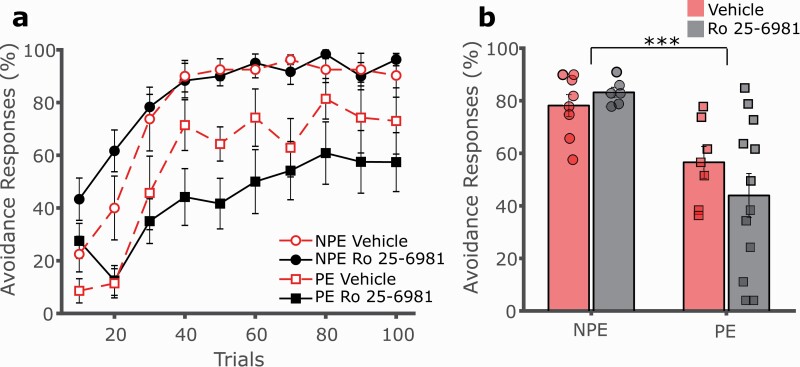
Ro25-6981 does not affect latent inhibition of 2-way avoidance. (A) The GluN2B receptor antagonist Ro25-6981 (6 mg/kg, i.p., black, solid lines) or vehicle (red, dashed lines) was administered systemically to rats prior to conditioning. Rats pre-exposed (PE, squares) to the CS avoided significantly less than non-preexposed (NPE, circles) controls irrespective of the drug treatment, with no effect on LI. (B) Overall avoidance percentage was significantly lower in PE vs NPE rats, with no effect of drug treatment. ***P < .001, error bars represent SEM.

To investigate the specific role of the NMDAR GluN2B subunit implicated in the induction of LTD, we systemically administered the GluN2B subunit-specific antagonist Ro25-6981 prior to conditioning. GluN2B receptor antagonism did not affect acquisition of 2-way avoidance in PE or NPE rats, indicating no significant effect of Ro25-6981 on LI (Figure 3A, B; 3-way mixed GLM, main effect of trials F(9,261) = 35.361, *P* < .001; main effect of exposure F(1,29) = 17.375, *P* < .001; main effect of drug F(1,29) = 0.274, *P* = .604; exposure by drug interaction F(1,29) = 1.470, *P* = .235). Thus, blocking GluN2B-dependent NMDAR signaling did not recapitulate the effect observed with the Tat-GluA2_3Y_ peptide. As before, escape responses declined over trials, with fewer escapes in NPE vs PE rats, with no effect of Ro25-6981 treatment (data not shown, main effect of trials F(9,261) = 22.375, *P* < .001; main effect of exposure F(1,29) = 8.062, *P* = .008; main effect of drug F(1,29) = 2.042, *P* = .164; exposure by drug interaction F(1,29) = 0.418, *P* = .523).

**Figure 4.**
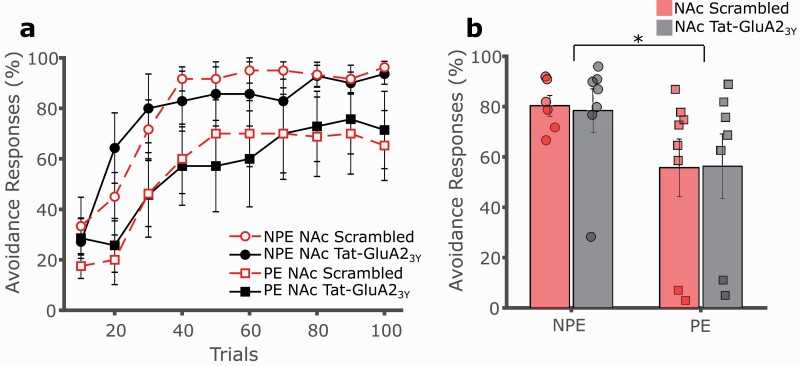
Nucleus accumbens (NAc) administration of Tat-GluA23Y does not affect latent inhibition (LI) of 2-way avoidance. (A) Tat-GluA23Y (0.5 μL/hemisphere, 22.5 pmol/0.5 μL, IC, black, solid lines) or scrambled control peptide (red, dashed lines) was administered into the NAc of rats prior to conditioning. Rats pre-exposed (PE, squares) to the CS avoided significantly less than non-preexposed (NPE, circles) controls irrespective of the drug treatment, with no effect on LI. (B) Overall avoidance percentage was significantly lower in PE vs NPE rats, with no effect of drug treatment. *P < .05, error bars represent SEM.

**Figure 5.**
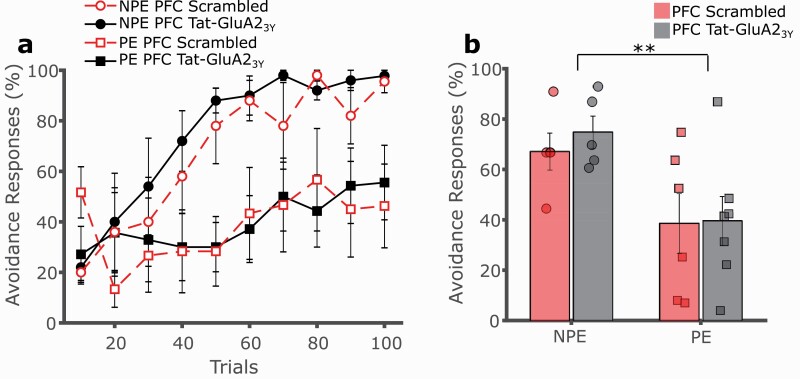
Medial prefrontal cortex (mPFC) administration of Tat-GluA23Y does not affect latent inhibition (LI) of 2-way avoidance. (A) Tat-GluA23Y (1.0 μL/hemisphere, 22.5 pmol/0.5 μL, IC, black, solid lines) or scrambled control peptide (red, dashed lines) was administered into the mPFC of rats prior to conditioning. Rats pre-exposed (PE, squares) to the CS avoided significantly less than non-preexposed (NPE, circles) controls irrespective of the drug treatment, with no effect on LI. (B) Overall avoidance percentage was significantly lower in PE vs NPE rats, with no effect of drug treatment. **P < .01, error bars represent SEM.

To determine a locus of action for the observed effect of systemic Tat-GluA2_3Y_ peptide administration, separate experiments were conducted in which the interference peptide was administered prior to conditioning via intracerebral microinjection to several key brain regions previously implicated in LI. Intra-NAc administration of the Tat-GluA2_3Y_ peptide did not alter the expression of LI relative to the control peptide (Figure 4A, B; 3-way mixed GLM, main effect of trials F(9,216) = 29.043, *P* < .001; main effect of exposure F(1,24) = 5.057, *P* = .034; main effect of drug F(1,24) = 0.004, *P* = .951; exposure by drug interaction F(1,24) = 0.016, *P* = .901). Similarly, intra-mPFC administration of the Tat-GluA2_3Y_ peptide did not affect expression of LI relative to the control peptide (Figure 5A, B; 3-way mixed GLM, main effect of trials F(9,171) = 10.494, *P* < .001; main effect of exposure F(1,19) = 10.857, *P* = .004; main effect of drug F(1,19) = 0.200, *P* = .660; exposure by drug interaction F(1,19) = 0.114, *P* = .740).

**Figure 6.**
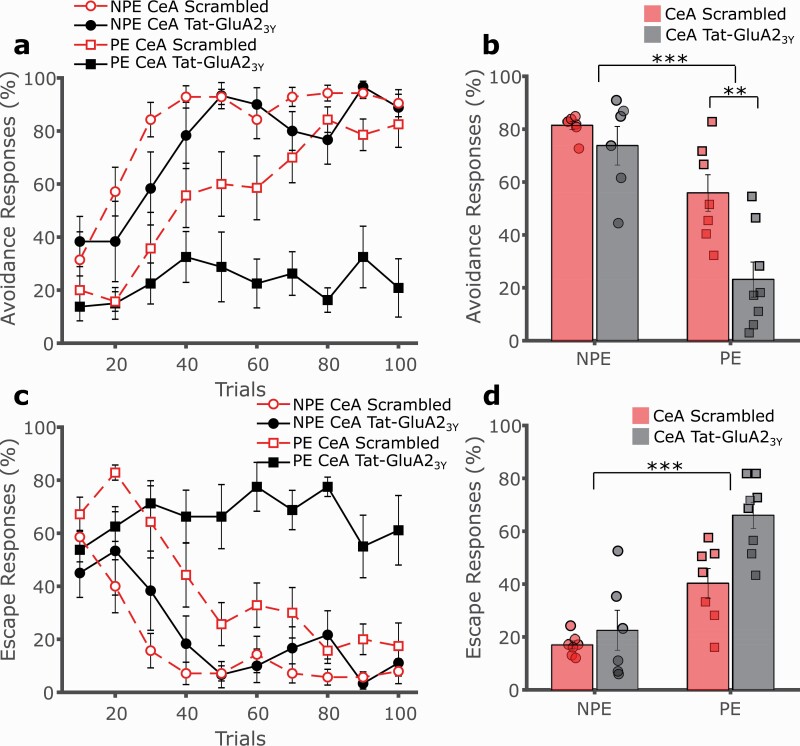
Central nucleus of the amygdala (CeA) administration of Tat-GluA23Y potentiates latent inhibition of 2-way avoidance. (A) Tat-GluA23Y (0.5 μL/hemisphere, 22.5 pmol/0.5 μL, IC, black, solid lines) or scrambled control peptide (red, dashed lines) was administered into the CeA of rats prior to conditioning. Rats pre-exposed (PE, squares) to the CS avoided significantly less than non-preexposed (NPE, circles) controls. Intra-CeA Tat-GluA23Y markedly strengthened the LI effect, by further impairing avoidance responding in PE rats (squares) relative to rats administered a scrambled control peptide (red, dashed lines), while having no effect in the NPE group (circles). (B) Overall avoidance percentage indicated a significant reduction of avoidances in intra-CeA Tat-GluA23Y-treated PE rats compared with the NPE group and the corresponding scrambled control, indicating a potentiation of LI. (C) Escape responses progressively declined over trials, with fewer escapes in NPE vs PE rats, while intra-CeA GluA23Y treatment tended to elevate escapes. (D) Overall escape responses percentage was significantly lower in PE vs NPE rats, while intra-CeA Tat-GluA23Y treatment increased escapes regardless of preexposure status. **P < .01, ***P < .001, error bars represent SEM.

Microinjection of Tat-GluA2_3Y_ peptide into the CeA significantly potentiated LI relative to the control peptide, as indicated by a profound impairment in the acquisition of 2-way avoidance in PE rats, compared with intact conditioning in their NPE counterparts and control PE rats (Figure 6A, B; 3-way mixed GLM, main effect of trials F(9,216) = 27.351, *P* < .001; main effect of exposure F(1,24) = 39.223, *P* < .001; main effect of drug F(1,24) = 11.152, *P* = .003; exposure by drug interaction F(1,24) = 4.375, *P* = .047; follow-up SME of drug in NPE F(1,24) = 0.726, *P* = .403; follow-up SME of drug in PE F(1,24) = 15.896, *P* = .001). Escapes declined progressively over time with fewer escapes in NPE vs PE rats (Figure 6C, D; main effect of trials F(9,216) = 13.877, *P* < .001; main effect of exposure F(1,24) = 40.996, *P* < .001), while Tat-GluA2_3Y_ treatment tended to elevate escapes in all groups, indicating that drug treatment did not impair perception of a noxious stimulus or locomotor activity (main effect of drug F(1,24) = 9.139, *P* = .006; exposure by drug interaction F(1,24) = 3.860, *P* = .061).

**Figure 7.**
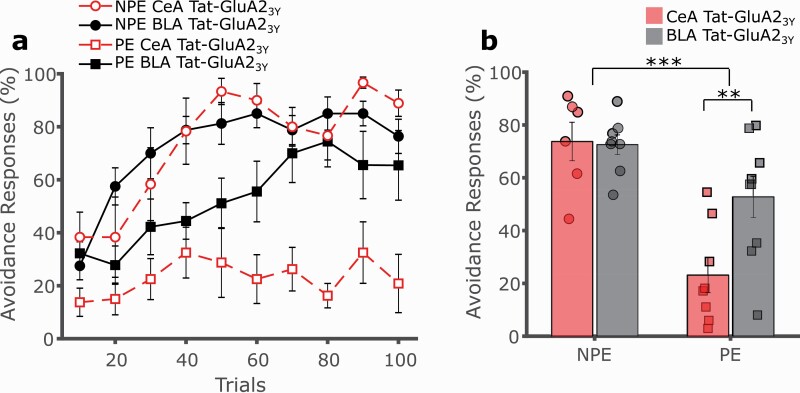
Basolateral amygdala administration of Tat-GluA23Y did not recapitulate the effect on LI observed following CeA administration. (A) Tat-GluA23Y (0.5 μL/hemisphere, 22.5 pmol/0.5 μL, IC) was administered into the CeA (red, dashed lines) or BLA (black, dashed lines) to rats prior to conditioning. Rats pre-exposed (PE, squares) to the CS avoided significantly less than non-preexposed (NPE, circles) controls. Intra-BLA Tat-GluA23Y administration had no effects on avoidance responding and thus failed to recapitulate the significant effect on LI observed following intra-CeA administration. This confirmed the CeA as the locus of action Tat-GluA23Y, excluding spill over to the BLA. (B) Overall avoidance percentage indicated a potentiation of LI in intra-CeA but not intra-BLA Tat-GluA23Y-treated rats. **P < .01, ***P < .001, error bars represent SEM.

To confirm this LI effect was mediated by drug action in the CeA and not due to spill over into the BLA, the Tat-GluA2_3Y_ peptide was administered directly into the BLA prior to conditioning in separate groups of PE and NPE rats. BLA administration of Tat-GluA2_3Y_ did not recapitulate the significant impairment of avoidance learning following CeA administration of Tat-GluA2_3Y_, confirming the CeA as the locus of action for the peptide (Figure 7; 3-way mixed GLM, main effect of trials F(9,243) = 19.200, *P* < .001; main effect of exposure F(1,27) = 27.396, *P* < .001; main effect of region F(1,27) = 4.459, *P* = .044; exposure by region interaction F(1,27) = 5.363, *P* = .028; follow-up SME of region in NPE F(1,27) = 0.019, *P* = .892; follow-up SME of region in PE F(1,27) = 10.954, *P* = .003).

## Discussion

As expected, pre-exposure to a nonreinforced stimulus impaired subsequent conditioning of this stimulus with reinforcement compared with the non-preexposed condition. The present LI paradigm involved strong CS pre-exposure (100 CS presentations over 2 days) and therefore produced robust LI. Although avoidance conditioning in PE rats was significantly lower than in NPE rats throughout the 100 trials, PE rats still displayed a learning curve. In stark contrast, systemic administration of the Tat-GluA2_3Y_ peptide completely blocked avoidance conditioning in PE rats, with no apparent learning curve. The Tat-GluA2_3Y_ peptide has been extensively validated in vivo across multiple brain regions as a specific inhibitor of clathrin-dependent endocytosis of GluA2-containing AMPARs, a critical step in LTD expression with no effect on basal synaptic transmission or LTP (Ahmadian et al., 2004; Brebner et al., 2005; Dalton et al., 2008; Yu et al., 2008; Ge et al., 2010; Dong et al., 2013).

Our results indicate that blocking LTD expression during the conditioning phase of a 2-way avoidance procedure potentiates LI by impairing avoidance learning to a pre-exposed CS. The absence of any disruptive effect of LTD blockade on conditioning with a novel CS in NPE rats excludes the possibility that this effect is due to a general impairment of associative learning per se or the capacity to detect and protect from aversive stimuli. This is consistent with intact fear conditioning under LTD blockade (Dalton et al., 2008, 2012) and extends this finding to active fear-conditioned avoidance. Thus, peptide-mediated LTD blockade during conditioning resulted in behavioral perseveration in the LI paradigm or an impaired capacity of PE rats to switch responding from the initial CS-no event association to the CS-US association, consistent with a role for LTD in mediating behavioral flexibility in response to changes in reinforcement contingency. Similarly, in previous studies of LTD blockade at the conditioning stage, fear conditioning is acquired but not extinguished (Kim et al., 2007; Dalton et al., 2008, 2012), and the initial hidden platform location on a water maze, but not its relocation, is learned (Nicholls et al., 2008; Kim et al., 2011; Dong et al., 2013). LTD is also required for natural forgetting of previous associations (Hardt et al., 2014; Migues et al., 2016).

Systemic blockade of LTD using the Tat-GluA2_3Y_ peptide produces similar effects on LI perseverance as dopamine blockers, NMDA antagonists, and cholinergic drugs (Weiner, 2003). As these drugs have known effects on synaptic plasticity, LTD represents a potential common mechanism by which such drugs may mediate their effect on LI, although other mechanisms may also be involved. Here we report significant treatment-induced strengthening of LI compared with strong LI in controls, whereas previous demonstrations of LI enhancement often involved weak or no LI in controls produced with fewer CS pre-exposure (Weiner, 2003), underscoring the robustness of our LTD blockade effect. As Tat-GluA2_3Y_ treatment entirely blocked conditioning in PE rats, an interesting question remains as to the effects of weaker pre-exposure protocols followed by LTD blockade in conditioning.

On the other hand, systemic administration of the GluN2B subunit-selective NMDAR antagonist, Ro25-6981, which specifically impairs GluN2B-dependent LTD in vivo (Liu et al., 2004; Fox et al., 2006; Ge et al., 2010), did not recapitulate the potentiated LI effect observed with the Tat-GluA2_3Y_ peptide. Considering that systemically induced blockade of glutamatergic transmission using the non-selective NMDA-R blocker MK-801 prior to conditioning potentiates LI (Gaisler-Salomon and Weiner, 2003; Weiner, 2003; Gaisler-Salomon et al., 2008), these results suggest that GluN2B-independent signaling may be involved in both the observed LTD effect on LI as well as the effect of glutamatergic drugs. The possibility remains that localized micro-injections of Ro25-6981 targeting GluN2B-dependent LTD within a specific region of interest are required to affect LI. Therefore, while our findings with the Tat-GluA2_3Y_ peptide provide compelling evidence for the role of LTD expression in mediating the observed effect on LI, future studies are warranted to establish which mechanisms of LTD initiation are involved.

The NAc plays a central role in LI as well as in the modulation of LI by dopaminergic drugs (Gray et al., 1997). Although systemic blockade of LTD using Tat-GluA2_3Y_ enhances LI similar to the effects of traditional neuroleptics, our microinjection experiments fail to implicate NAc LTD in the LI facilitation. Our microinjections targeted the NAc core, where lesions enhance LI, with limited spread into the shell, where lesions disrupt LI. It is possible that incomplete LTD blockade in the shell accounts for the failure of our intra-NAc microinjections to modulate LI (Weiner et al., 1996; Jongen-Rêlo et al., 2002; Gal et al., 2005). Brain regions that provide afferent projections to the NAc are also implicated in LI, including the mPFC (George et al., 2010; Lingawi et al., 2016) and BLA (Weiner et al., 1995; Coutureau et al., 2001; Schiller and Weiner, 2004). We failed to observe an effect of intra-mPFC injection of the Tat-GluA2_3Y_ peptide on LI. We targeted the dorsal aspect of mPFC (anterior cingulate-prelimbic border), which, combined with the volume delivered (0.75 µL), likely spared ventral mPFC (infralimbic cortex). As ventral mPFC lesions may affect LI (George et al., 2010; Lingawi et al., 2016), a potential role for LTD in specific subregions of the mPFC remains plausible and warrants further investigation.

Administration of the GluA2_3Y_ peptide to block LTD within the intra-CeA recapitulated the robust conditioning impairment in CS-PE groups following its systemic administration. This finding, in turn, suggests that LTD in the central amygdala may be a key mediator of cognitive flexibility that enables previously irrelevant stimuli to acquire significance through reinforcement. The CeA is the principal output structure of the amygdala and is required for expression of fear and defensive behaviors to threatening stimuli (LeDoux and Phillips, 1992; Gozzi et al., 2010; Haubensak et al., 2010; Steinberg et al., 2020). Accumulating evidence supports a role for the CeA in both aversive and appetitive emotional learning and as a critical site of plasticity in some forms of Pavlovian learning (Samson and Paré, 2005; Samson et al., 2005; Tillman et al., 2018; Steinberg et al., 2020).

The CeA consists primarily of GABAergic neurons organized into an intricate microcircuit that controls the expression of conditioned fear as an active or passive response (Ciocchi et al., 2010; Gozzi et al., 2010; Haubensak et al., 2010; Li et al., 2013; Fadok et al., 2017; Yu et al., 2017). As this GABAergic neuronal circuitry does not undergo GluA2-dependent LTD, we propose that an excitatory input to a subpopulation of CeA neurons is initially potentiated during CS pre-exposure and must then undergo LTD during subsequent conditioning. One candidate might be threat-encoding excitatory inputs from the BLA that synapse onto CeA interneurons, which in turn initiate behavioral responses via inhibitory action onto downstream targets (Babaev et al., 2018). Consideration should also be given to a subpopulation of PKC+ GABAergic cells within the lateral region of the CeA as a possible target of these inputs, as these neurons cease to display action potentials during acquisition of conditioned fear (Haubensak et al., 2010).

Our data suggest that PE rats in the control and ineffective treatment conditions express both the CS-no event and CS-US associations, whereas PE rats treated with either systemic or intra-CeA administration of the Tat-GluA2_3Y_ peptide remain exclusively under the behavioral control of the CS-no-event association. Building on Weiner’s competition theory of LI, which emphasizes involvement of opposing processes between 2 conflicting associations competing for behavioral expression/retrieval, LTD in the CeA may mediate active inhibition of neural circuitry that encodes the original stimulus-no event association. This, in turn, would permit the formation of a new association between this CS and reinforcement. In the absence of such an LTD mechanism to inhibit a prior association with the same CS, as in the presence of the interference peptide, the initial association would effectively compete with the establishment of a new more relevant acccociation. Through its interaction with the midbrain dopamine system (Ahn and Phillips, 2002; Steinberg et al., 2020), the CeA has also been linked to enhanced attention to cues following changes in their predictive value (Holland and Gallagher, 1993a, 2006; Fudge and Haber, 2000; Lee et al., 2006; Tillman et al., 2018). Accordingly, in the LI paradigm, CeA LTD may also prevent enhancement of attention to changes in stimulus salience required for successful conditioning, consistent with the acquisition-failure theory (Lubow et al., 1976; Pearce and Hall, 1980). Thus, modulation of synaptic plasticity in the CeA may have far-reaching consequences for behavioral responses to threat under changing circumstances.

The LI paradigm used here involves the acquisition of an active response to an aversive contingency. LI effects are observed in both passive fear paradigms and appetitive paradigms; therefore, further studies are required to determine whether the present finding generalizes to these circumstances. Although the CeA is implicated in certain forms of conditioned appetitive behavior (Hall et al., 2001; Holland and Gallagher, 2003; Steinberg et al., 2020), neurotoxic lesions of the CeA failed to modulate LI in an appetitive procedure (Holland and Gallagher, 1993b). As the neural circuitry mediating conditioned fear differs from that subserving the effects of LI on appetitive learning, it remains to be determined whether disruption of LTD in the appropriate synapses would affect cognitive flexibility required for adaptation of appetitive learning.

Interest in LI gains added significance through its link to schizophrenia and related disorders, giving rise to the “2-headed” LI model of schizophrenia (Weiner, 2003; Weiner and Arad, 2009). On one hand, LI deficits are observed in preclinical models of schizophrenia symptoms, canonically induced by amphetamine administration, with disrupted context processing highlighted as a key feature (Weiner, 1990; Gray et al., 1991). Similarly, the clinical data generally support reduced LI in acute, non-/recently medicated, positive-symptom schizophrenic patients compared with healthy controls (Lubow, 2010; Lubow and Weiner, 2010b). On the other hand, LI perseveration, or the inability to update behavior based on changing relationships between stimuli and outcomes, is a core disturbance in chronic schizophrenia (Gal et al., 2009; Granger et al., 2016), implying that impaired LTD may be involved. Furthermore, the abnormally persistent LI observed in chronic schizophrenia patients may account for their impaired attentional set shifting, a form of disrupted cognitive flexibility (Weiner, 2003). LI abnormalities in schizophrenia appear to depend on the state of the disorder (acute vs chronic), providing clinical context for our findings of potentiated LI following LTD blockade, with implications for the positive, negative, and cognitive symptoms of schizophrenia (Weiner, 2003).

Finally, the present findings are consistent with preclinical and clinical studies that link dysfunction within the glutamatergic system and aberrant synaptic plasticity to schizophrenia. As mentioned, NMDA receptor antagonists, which induce persistent LI and model aspects of schizophrenia in rodents, support the hypo-glutamatergic hypothesis and the presence of impaired plasticity in schizophrenia (Gaisler-Salomon and Weiner, 2003; Weiner, 2003; Gaisler-Salomon et al., 2008). Consistent with this hypothesis, noninvasive human brain stimulation studies report reduced LTP and LTD in the motor cortex of schizophrenia patients (Hasan et al., 2012; Bhandari et al., 2016). Modulation of LTD provides a potent mechanism for targeting aberrant LI and deserves consideration as a potential therapeutic strategy for key symptoms in schizophrenia arising from abnormal stimulus processing. In summary, the present findings have relevance both for our mechanistic understanding of LI and its alteration in pathological states such as schizophrenia, while further elucidating the role of LTD in learning and memory.

## Supplementary Material

pyab011_suppl_Supplementary_Materials

## Statement of Interest

Y.T.W. and A.G.P. declare a patent related to glutamate receptor function (A Peptide that Specifically Blocks Regulated AMPA Receptor Endocytosis and Hippocampal CA1 Long-term Depression; European 04789721.0, and United States 13/066,700). A.G.P. also declares a patent related to the use of d-govadine to enhance dopamine function in the prefrontal cortex (Tetrahydroprotoberbine compounds and uses thereof in the treatment of neurological, psychiatric and neurodegenerative diseases; US20150306092). A.G.P. also holds shares in Resilience Biosciences Inc., Canada. Other authors report no other biomedical financial interests or potential conflicts of interest.

## References

[CIT0001] Ahmadian G , Ju W, Liu L, Wyszynski M, Lee SH, Dunah AW, Taghibiglou C, Wang Y, Lu J, Wong TP, Sheng M, Wang YT (2004) Tyrosine phosphorylation of GluR2 is required for insulin-stimulated AMPA receptor endocytosis and LTD. EMBO J 23:1040–1050. 14976558 10.1038/sj.emboj.7600126 PMC380981

[CIT0002] Ahn S , Phillips AG (2002) Modulation by central and basolateral amygdalar nuclei of dopaminergic correlates of feeding to satiety in the rat nucleus accumbens and medial prefrontal cortex. J Neurosci 22:10958–10965. 12486191 10.1523/JNEUROSCI.22-24-10958.2002 PMC6758436

[CIT0003] Babaev O , Piletti Chatain C, Krueger-Burg D (2018) Inhibition in the amygdala anxiety circuitry. Exp Mol Med 50:1–16. 10.1038/s12276-018-0063-8 PMC5938054 29628509

[CIT0004] Bhandari A , Voineskos D, Daskalakis ZJ, Rajji TK, Blumberger DM (2016) A review of impaired neuroplasticity in schizophrenia investigated with non-invasive brain stimulation. Front Psychiatry 7:45. 27065890 10.3389/fpsyt.2016.00045 PMC4810231

[CIT0005] Brebner K , Wong TP, Liu L, Liu Y, Campsall P, Gray S, Phelps L, Phillips AG, Wang YT (2005) Nucleus accumbens long-term depression and the expression of behavioral sensitization. Science 310:1340–1343. 16311338 10.1126/science.1116894

[CIT0006] Ciocchi S , Herry C, Grenier F, Wolff SB, Letzkus JJ, Vlachos I, Ehrlich I, Sprengel R, Deisseroth K, Stadler MB, Müller C, Lüthi A (2010) Encoding of conditioned fear in central amygdala inhibitory circuits. Nature 468:277–282. 21068837 10.1038/nature09559

[CIT0007] Citri A , Malenka RC (2008) Synaptic plasticity: multiple forms, functions, and mechanisms. Neuropsychopharmacology 33:18–41. 17728696 10.1038/sj.npp.1301559

[CIT0008] Collingridge GL , Peineau S, Howland JG, Wang YT (2010) Long-term depression in the CNS. Nat Rev Neurosci 11:459–473. 20559335 10.1038/nrn2867

[CIT0009] Coutureau E , Blundell PJ, Killcross S (2001) Basolateral amygdala lesions disrupt latent inhibition in rats. Brain Res Bull 56:49–53. 11604248 10.1016/s0361-9230(01)00592-5

[CIT0010] Dalton GL , Wang YT, Floresco SB, Phillips AG (2008) Disruption of AMPA receptor endocytosis impairs the extinction, but not acquisition of learned fear. Neuropsychopharmacology 33:2416–2426. 18046303 10.1038/sj.npp.1301642

[CIT0011] Dalton GL , Wu DC, Wang YT, Floresco SB, Phillips AG (2012) NMDA GluN2A and GluN2B receptors play separate roles in the induction of LTP and LTD in the amygdala and in the acquisition and extinction of conditioned fear. Neuropharmacology 62:797–806. 21925518 10.1016/j.neuropharm.2011.09.001

[CIT0012] Dong Z , Bai Y, Wu X, Li H, Gong B, Howland JG, Huang Y, He W, Li T, Wang YT (2013) Hippocampal long-term depression mediates spatial reversal learning in the Morris water maze. Neuropharmacology 64:65–73. 22732443 10.1016/j.neuropharm.2012.06.027

[CIT0013] Fadok JP , Krabbe S, Markovic M, Courtin J, Xu C, Massi L, Botta P, Bylund K, Müller C, Kovacevic A, Tovote P, Lüthi A (2017) A competitive inhibitory circuit for selection of active and passive fear responses. Nature 542:96–100. 28117439 10.1038/nature21047

[CIT0014] Floresco SB (2015) Noradrenaline and dopamine: sharing the workload. Trends Neurosci 38:465–467. 26183088 10.1016/j.tins.2015.07.001

[CIT0015] Fox CJ , Russell KI, Wang YT, Christie BR (2006) Contribution of NR2A and NR2B NMDA subunits to bidirectional synaptic plasticity in the hippocampus in vivo. Hippocampus 16:907–915. 17024679 10.1002/hipo.20230

[CIT0016] Fudge JL , Haber SN (2000) The central nucleus of the amygdala projection to dopamine subpopulations in primates. Neuroscience 97:479–494. 10828531 10.1016/s0306-4522(00)00092-0

[CIT0017] Gaisler-Salomon I , Diamant L, Rubin C, Weiner I (2008) Abnormally persistent latent inhibition induced by MK801 is reversed by risperidone and by positive modulators of NMDA receptor function: differential efficacy depending on the stage of the task at which they are administered. Psychopharmacology 196:255–267. 17928997 10.1007/s00213-007-0960-3

[CIT0018] Gaisler-Salomon I , Weiner I (2003) Systemic administration of MK-801 produces an abnormally persistent latent inhibition which is reversed by clozapine but not haloperidol. Psychopharmacology 166:333–342. 12599023 10.1007/s00213-002-1311-z

[CIT0019] Gal G , Schiller D, Weiner I (2005) Latent inhibition is disrupted by nucleus accumbens shell lesion but is abnormally persistent following entire nucleus accumbens lesion: the neural site controlling the expression and disruption of the stimulus preexposure effect. Behav Brain Res 162:246–255. 15970218 10.1016/j.bbr.2005.03.019

[CIT0020] Gal G , Barnea Y, Biran L, Mendlovic S, Gedi T, Halavy M, Feldon J, Fennig S, Levkovitz Y (2009) Enhancement of latent inhibition in patients with chronic schizophrenia. Behav Brain Res 197:1–8. 18793680 10.1016/j.bbr.2008.08.023

[CIT0021] Ge Y , Dong Z, Bagot RC, Howland JG, Phillips AG, Wong TP, Wang YT (2010) Hippocampal long-term depression is required for the consolidation of spatial memory. Proc Natl Acad Sci USA 107:16697–16702. 20823230 10.1073/pnas.1008200107 PMC2944752

[CIT0022] George DN , Duffaud AM, Pothuizen HH, Haddon JE, Killcross S (2010) Lesions to the ventral, but not the dorsal, medial prefrontal cortex enhance latent inhibition. Eur J Neurosci 31:1474–1482. 20384772 10.1111/j.1460-9568.2010.07178.x

[CIT0023] Gozzi A , Jain A, Giovannelli A, Giovanelli A, Bertollini C, Crestan V, Schwarz AJ, Tsetsenis T, Ragozzino D, Gross CT, Bifone A (2010) A neural switch for active and passive fear. Neuron 67:656–666. 20797541 10.1016/j.neuron.2010.07.008

[CIT0024] Granger KT , Moran PM, Buckley MG, Haselgrove M (2016) Enhanced latent inhibition in high schizotypy individuals. Pers Indiv Differ 91:31–39.

[CIT0025] Gray JA , Feldon J, Rawlins JNP, Hemsley DR, Smith AD (1991) The neuropsychology of schizophrenia. Behav Brain Sci 14:1–20.

[CIT0026] Gray JA , Moran PM, Grigoryan G, Peters SL, Young AM, Joseph MH (1997) Latent inhibition: the nucleus accumbens connection revisited. Behav Brain Res 88:27–34. 9401705 10.1016/s0166-4328(97)02313-9

[CIT0027] Hall J , Parkinson JA, Connor TM, Dickinson A, Everitt BJ (2001) Involvement of the central nucleus of the amygdala and nucleus accumbens core in mediating Pavlovian influences on instrumental behaviour. Eur J Neurosci 13:1984–1992. 11403692 10.1046/j.0953-816x.2001.01577.x

[CIT0028] Hardt O , Nader K, Wang YT (2014) GluA2-dependent AMPA receptor endocytosis and the decay of early and late long-term potentiation: possible mechanisms for forgetting of short- and long-term memories. Philos Trans R Soc Lond B Biol Sci 369:20130141. 24298143 10.1098/rstb.2013.0141 PMC3843873

[CIT0029] Hasan A , Nitsche MA, Herrmann M, Schneider-Axmann T, Marshall L, Gruber O, Falkai P, Wobrock T (2012) Impaired long-term depression in schizophrenia: a cathodal tDCS pilot study. Brain Stimul 5:475–483. 21945231 10.1016/j.brs.2011.08.004

[CIT0030] Haubensak W , Kunwar PS, Cai H, Ciocchi S, Wall NR, Ponnusamy R, Biag J, Dong HW, Deisseroth K, Callaway EM, Fanselow MS, Lüthi A, Anderson DJ (2010) Genetic dissection of an amygdala microcircuit that gates conditioned fear. Nature 468:270–276. 21068836 10.1038/nature09553 PMC3597095

[CIT0031] Holland PC , Gallagher M (1993a) Effects of amygdala central nucleus lesions on blocking and unblocking. Behav Neurosci 107:235–245. 8484889 10.1037//0735-7044.107.2.235

[CIT0032] Holland PC , Gallagher M (1993b) Amygdala central nucleus lesions disrupt increments, but not decrements, in conditioned stimulus processing. Behav Neurosci 107:246–253. 8484890 10.1037//0735-7044.107.2.246

[CIT0033] Holland PC , Gallagher M (2003) Double dissociation of the effects of lesions of basolateral and central amygdala on conditioned stimulus-potentiated feeding and Pavlovian-instrumental transfer. Eur J Neurosci 17:1680–1694. 12752386 10.1046/j.1460-9568.2003.02585.x

[CIT0034] Holland PC , Gallagher M (2006) Different roles for amygdala central nucleus and substantia innominata in the surprise-induced enhancement of learning. J Neurosci 26:3791–3797. 16597732 10.1523/JNEUROSCI.0390-06.2006 PMC6674125

[CIT0035] Howland JG , Wang YT (2008) Synaptic plasticity in learning and memory: stress effects in the hippocampus. Prog Brain Res 169:145–158. 18394472 10.1016/S0079-6123(07)00008-8

[CIT0036] Jongen-Rêlo AL , Kaufmann S, Feldon J (2002) A differential involvement of the shell and core subterritories of the nucleus accumbens of rats in attentional processes. Neuroscience 111:95–109. 11955715 10.1016/s0306-4522(01)00521-8

[CIT0037] Kim JI , et al. (2011) PI3Kγ is required for NMDA receptor-dependent long-term depression and behavioral flexibility. Nat Neurosci 14:1447–1454. 22019731 10.1038/nn.2937

[CIT0038] Kim J , Lee S, Park K, Hong I, Song B, Son G, Park H, Kim WR, Park E, Choe HK, Kim H, Lee C, Sun W, Kim K, Shin KS, Choi S (2007) Amygdala depotentiation and fear extinction. Proc Natl Acad Sci USA 104:20955–20960. 18165656 10.1073/pnas.0710548105 PMC2409248

[CIT0039] LeDoux JE , Phillips R (1992) Differential contribution of amygdala and hippocampus to cued and contextual fear conditioning. Behav Neurosci 106:274–285. 1590953 10.1037//0735-7044.106.2.274

[CIT0040] Lee HJ , Youn JM, O MJ, Gallagher M, Holland PC (2006) Role of substantia nigra-amygdala connections in surprise-induced enhancement of attention. J Neurosci 26:6077–6081. 16738251 10.1523/JNEUROSCI.1316-06.2006 PMC6675230

[CIT0041] Li H , Penzo MA, Taniguchi H, Kopec CD, Huang ZJ, Li B (2013) Experience-dependent modification of a central amygdala fear circuit. Nat Neurosci 16:332–339. 23354330 10.1038/nn.3322 PMC3581751

[CIT0042] Lingawi NW , Westbrook RF, Laurent V (2016) Extinction and latent inhibition involve a similar form of inhibitory learning that is stored in and retrieved from the infralimbic cortex. Cereb Cortex 27:5547–5556. 10.1093/cercor/bhw322 27797830

[CIT0043] Liu L , Wong TP, Pozza MF, Lingenhoehl K, Wang Y, Sheng M, Auberson YP, Wang YT (2004) Role of NMDA receptor subtypes in governing the direction of hippocampal synaptic plasticity. Science 304:1021–1024. 15143284 10.1126/science.1096615

[CIT0044] Lubow RE (1973) Latent inhibition. Psychol Bull 79:398–407. 4575029 10.1037/h0034425

[CIT0045] Lubow RE (2010) Latent inhibition and schizophrenia: the ins and outs of context. In: Latent inhibition (Lubow RE, Weiner I, eds), pp 500–528. Cambridge: Cambridge University Press.

[CIT0046] Lubow RE , Schnur P, Rifkin B (1976) Latent inhibition and conditioned attention theory. J Exp Psychol Anim Behav Process 2:163–174.

[CIT0047] Lubow RE , Weiner I (2010a) Latent inhibition: cognition, neuroscience and applications to schizophrenia (Lubow RE, Weiner I, eds). Cambridge: Cambridge University Press.

[CIT0048] Lubow RE , Weiner I (2010b) Issues in latent inhibition research and theory: an overview. In: Latent inhibition (Lubow RE, Weiner I, eds), pp 531–557. Cambridge: Cambridge University Press.

[CIT0049] Migues PV , Liu L, Archbold GE, Einarsson EÖ, Wong J, Bonasia K, Ko SH, Wang YT, Hardt O (2016) Blocking synaptic removal of GluA2-containing AMPA receptors prevents the natural forgetting of long-term memories. J Neurosci 36:3481–3494. 27013677 10.1523/JNEUROSCI.3333-15.2016 PMC6601735

[CIT0050] Nicholls RE , Alarcon JM, Malleret G, Carroll RC, Grody M, Vronskaya S, Kandel ER (2008) Transgenic mice lacking NMDAR-dependent LTD exhibit deficits in behavioral flexibility. Neuron 58:104–117. 18400167 10.1016/j.neuron.2008.01.039

[CIT0051a] Paxinos G , Watson C (2013) The rat brain in stereotaxic coordinates. 7th Ed, p472. Amsterdam: Academic Press.

[CIT0051] Pearce JM , Hall G (1980) A model for Pavlovian learning: variations in the effectiveness of conditioned but not of unconditioned stimuli. Psychol Rev 87:532–552. 7443916

[CIT0052] Pothuizen HH , Jongen-Rêlo AL, Feldon J, Yee BK (2005) Double dissociation of the effects of selective nucleus accumbens core and shell lesions on impulsive-choice behaviour and salience learning in rats. Eur J Neurosci 22:2605–2616. 16307603 10.1111/j.1460-9568.2005.04388.x

[CIT0053] Samson RD , Duvarci S, Paré D (2005) Synaptic plasticity in the central nucleus of the amygdala. Rev Neurosci 16:287–302. 16519006 10.1515/revneuro.2005.16.4.287

[CIT0054] Samson RD , Paré D (2005) Activity-dependent synaptic plasticity in the central nucleus of the amygdala. J Neurosci 25:1847–1855. 15716421 10.1523/JNEUROSCI.3713-04.2005 PMC6725937

[CIT0055] Schiller D , Weiner I (2004) Lesions to the basolateral amygdala and the orbitofrontal cortex but not to the medial prefrontal cortex produce an abnormally persistent latent inhibition in rats. Neuroscience 128:15–25. 15450350 10.1016/j.neuroscience.2004.06.020

[CIT0056] Schiller D , Zuckerman L, Weiner I (2006) Abnormally persistent latent inhibition induced by lesions to the nucleus accumbens core, basolateral amygdala and orbitofrontal cortex is reversed by clozapine but not by haloperidol. J Psychiatr Res 40:167–177. 15882871 10.1016/j.jpsychires.2005.03.002

[CIT0057] Schwarze SR , Ho A, Vocero-Akbani A, Dowdy SF (1999) In vivo protein transduction: delivery of a biologically active protein into the mouse. Science 285:1569–1572. 10477521 10.1126/science.285.5433.1569

[CIT0058] Steinberg EE , Gore F, Heifets BD, Taylor MD, Norville ZC, Beier KT, Földy C, Lerner TN, Luo L, Deisseroth K, Malenka RC (2020) Amygdala-midbrain connections modulate appetitive and aversive learning. Neuron 106:1026–1043.e9. 32294466 10.1016/j.neuron.2020.03.016

[CIT0059] Takeuchi T , Duszkiewicz AJ, Morris RG (2014) The synaptic plasticity and memory hypothesis: encoding, storage and persistence. Philos Trans R Soc Lond B Biol Sci 369:20130288. 24298167 10.1098/rstb.2013.0288 PMC3843897

[CIT0060] Tillman RM , Stockbridge MD, Nacewicz BM, Torrisi S, Fox AS, Smith JF, Shackman AJ (2018) Intrinsic functional connectivity of the central extended amygdala. Hum Brain Mapp 39:1291–1312. 29235190 10.1002/hbm.23917 PMC5807241

[CIT0061] Weiner I (1990) Neural substrates of latent inhibition: the switching model. Psychol Bull 108:442–461. 1980149 10.1037/0033-2909.108.3.442

[CIT0062] Weiner I (2003) The “two-headed” latent inhibition model of schizophrenia: modeling positive and negative symptoms and their treatment. Psychopharmacology 169:257–297. 12601500 10.1007/s00213-002-1313-x

[CIT0063] Weiner I , Arad M (2009) Using the pharmacology of latent inhibition to model domains of pathology in schizophrenia and their treatment. Behav Brain Res 204:369–386. 19433114 10.1016/j.bbr.2009.05.004

[CIT0064] Weiner I , Feldon J (1997) The switching model of latent inhibition: an update of neural substrates. Behav Brain Res 88:11–25. 9401704 10.1016/s0166-4328(97)02314-0

[CIT0065] Weiner I , Gal G, Feldon J (1999) Disrupted and undisruptable latent inhibition following shell and core lesions. Ann N Y Acad Sci 877:723–727. 10415692 10.1111/j.1749-6632.1999.tb09310.x

[CIT0066] Weiner I , Gal G, Rawlins JN, Feldon J (1996) Differential involvement of the shell and core subterritories of the nucleus accumbens in latent inhibition and amphetamine-induced activity. Behav Brain Res 81:123–133. 8950008 10.1016/s0166-4328(96)00051-4

[CIT0067] Weiner I , Tarrasch R, Feldon J (1995) Basolateral amygdala lesions do not disrupt latent inhibition. Behav Brain Res 72:73–81. 8788859 10.1016/0166-4328(96)00056-3

[CIT0068] Yu K , Ahrens S, Zhang X, Schiff H, Ramakrishnan C, Fenno L, Deisseroth K, Zhao F, Luo MH, Gong L, He M, Zhou P, Paninski L, Li B (2017) The central amygdala controls learning in the lateral amygdala. Nat Neurosci 20:1680–1685. 29184202 10.1038/s41593-017-0009-9 PMC5755715

[CIT0069] Yu SY , Wu DC, Liu L, Ge Y, Wang YT (2008) Role of AMPA receptor trafficking in NMDA receptor-dependent synaptic plasticity in the rat lateral amygdala. J Neurochem 106:889–899. 18466342 10.1111/j.1471-4159.2008.05461.x

