# Supplementary material for: Disruption of Long-Term Depression Potentiates Latent Inhibition: Key Role for Central Nucleus of the Amygdala
Source: Int J Neuropsychopharmacol. 2021 Mar 8;24(7):580–91. doi: 10.1093/ijnp/pyab011 (PMC8299826; doi:10.1093/ijnp/pyab011)
Supplement: pyab011_suppl_Supplementary_Materials [file pyab011_suppl_supplementary_materials.docx]

**Ashby et al., *IJNP,* 2021**

**Disruption of long-term depression potentiates latent inhibition: Key role for central nucleus of the amygdala**

**Supplementary Information**


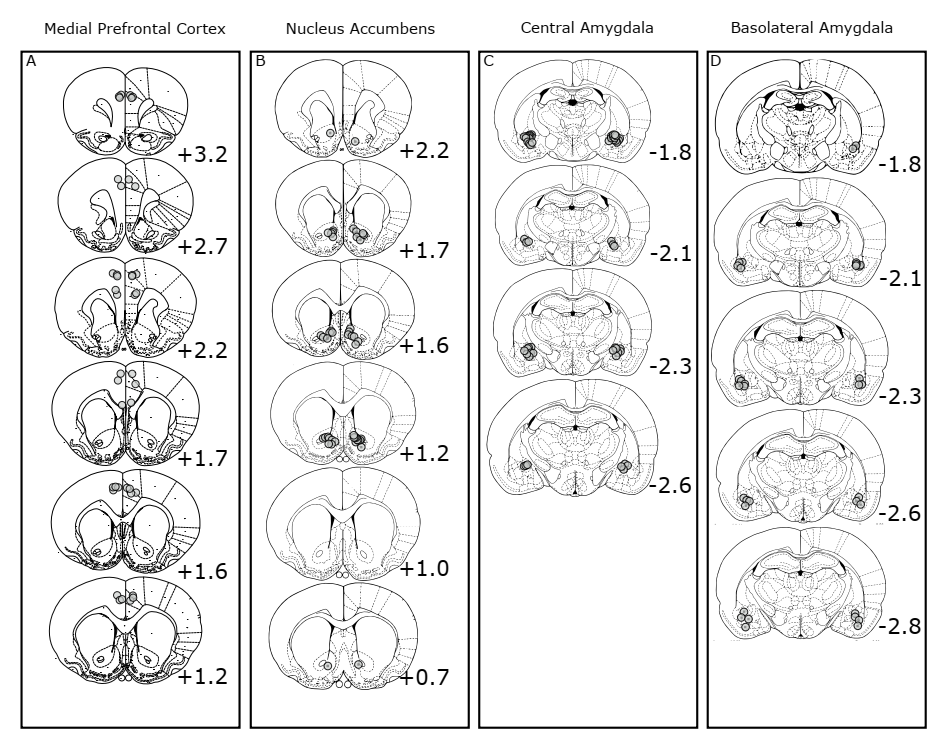


## **Supplementary Figure 1. Histology of the intracerebral administration of Tat-GluA2_3Y_ or Tat-GluA2_Sc_** **in the *a.* medial prefrontal cortex, *b.* nucleus accumbens, *c.* central or *d.* basolateral amygdala.**
